# Blood urea nitrogen and cardiovascular disease risk: Evidence from the CHARLS cohort study

**DOI:** 10.1097/MD.0000000000045722

**Published:** 2025-11-14

**Authors:** Renyu Jiang, Qi Wang, Jinyan Chen, Yong Hu

**Affiliations:** a First School of Clinical Medicine, Shandong University of Traditional Chinese Medicine, Jinan, China; b Department of Nephropathy, Qingdao Hospital of Traditional Chinese Medicine, Qingdao, China; c Department of Geriatrics, Qingdao Hospital of Traditional Chinese Medicine, Qingdao, China.

**Keywords:** biomarkers, blood urea nitrogen, cardiovascular disease, middle-aged and older adults, screening and prevention, China Health and Retirement Longitudinal Study

## Abstract

Blood urea nitrogen (BUN) is an important biomarker reflecting renal function and has broad potential applications in predicting cardiovascular disease (CVD) and maintaining the heart-kidney balance. Therefore, this study aims to investigate the association between BUN levels and the incidence of new-onset cardiovascular disease in the general population aged 45 years and older, using data from a prospective cohort study. This study utilized data from waves 1 to 5 of the China Health and Retirement Longitudinal Study, including a total of 9886 participants aged 45 years and older. Specifically, a multivariable logistic regression model was applied to analyze the relationship between BUN levels and new-onset CVD. In addition, restricted cubic spline (RCS) analyses were conducted to assess potential nonlinear associations. During a follow-up period of up to 9 years, 1055 participants (10.67%) developed cardiovascular disease. Compared with individuals in the lowest quartile (Q1), those in the highest quartile (Q4) of BUN levels had a relatively lower risk of CVD (OR  = 0.83; 95% CI: 0.69–1.01), although the result was not statistically significant. RCS analysis did not reveal a clear dose–response relationship (*P*-overall = .0238; *P*-nonlinear = .0879), but a potential risk threshold was observed around a BUN level of 15.13 mg/dL. These findings indicate a potential association between BUN levels and new-onset CVD risk in adults aged 45 years and above. While this suggests that BUN might serve as an independent predictor, further validation is needed to establish its clinical utility. These results contribute to the growing body of evidence regarding cardiovascular risk factors and may inform future research on early screening and prevention strategies.

## 1. Introduction

With the acceleration of population aging in China, the proportion of elderly individuals is increasing year by year, and advancing age has become one of the major risk factors for cardiovascular disease (CVD).^[1]^ Against this background, the 2023 Report on Cardiovascular Health and Disease in China indicates that CVD has become the leading cause of death among both urban and rural residents, with the number of affected individuals reaching 330 million.^[2]^ Globally, the number of people living with CVD continues to rise, currently exceeding 523 million.^[3]^ Although advances in medical care and improved economic conditions have led to a significantly higher survival rate among CVD patients, the overall prevalence of chronic diseases has also increased. This trend poses more complex and long-term challenges for health management.^[3]^ The burden of CVD varies markedly across regions and populations. Compared to other regions of the world, Asian countries exhibit higher age-adjusted mortality rates due to CVD.^[4]^ Given that Asia currently has a population of 4.8 billion, accounting for 60% of the global population, this disparity in mortality rates has a more pronounced impact on the global burden of disease.^[5]^ In China, factors such as uneven regional development and a unique population structure contribute to the continuing rise in CVD burden.^[6]^ To effectively curb this growing trend, early intervention is of critical importance. Identifying efficient and sensitive biomarkers represents a key step toward early screening and precision prevention of CVD. Previous studies have highlighted the predictive value of high-sensitivity C-reactive protein and certain genetic polymorphisms in identifying individuals at risk of developing CVD.^[7,8]^ Building on this foundation, further exploration of other potential clinical biomarkers may enhance the effectiveness of early screening and targeted intervention strategies, particularly in high-risk middle-aged and elderly populations.

Blood urea nitrogen (BUN) is a metabolic byproduct generated in the liver during protein catabolism and excreted by the kidneys.^[9]^ Because BUN levels reflect the dynamic balance between urea production and renal excretory capacity, they are widely used in clinical practice as an important biomarker for evaluating renal function and the severity of kidney injury.^[10]^ In recent years, a growing body of evidence has revealed a close and bidirectional relationship between renal function and CVD. Impaired renal excretion can lead to the accumulation of metabolic waste, which may adversely affect cardiovascular function. In addition, the kidneys and cardiovascular system jointly regulate blood pressure, fluid balance, and metabolic homeostasis. As such, renal dysfunction is not only a frequent comorbidity of CVD but may also act as a precipitating factor in its development.^[11–13]^ Given this physiological interplay, BUN – an indicator of renal function – has been proposed as a potential marker associated with CVD risk. Several studies have reported a positive association between elevated BUN levels and increased CVD risk, particularly among middle-aged and older adults.^[14]^ However, findings have been inconsistent. Stratified analyses in some studies suggest a possible inverse association in specific age groups, implying that age may moderate the relationship between BUN and CVD. Nevertheless, systematic investigations into this association remain limited, and the underlying mechanisms are not yet fully understood. To address this gap, the present study aims to explore the relationship between BUN levels and the risk of cardiovascular disease among Chinese adults aged 45 years and older using data from the China Health and Retirement Longitudinal Study (CHARLS). Employing a prospective cohort design, this study seeks to provide epidemiological evidence and theoretical support for early screening and precision intervention in this population.

## 2. Materials and methods

### 2.1. Data source

**Figure 1.**
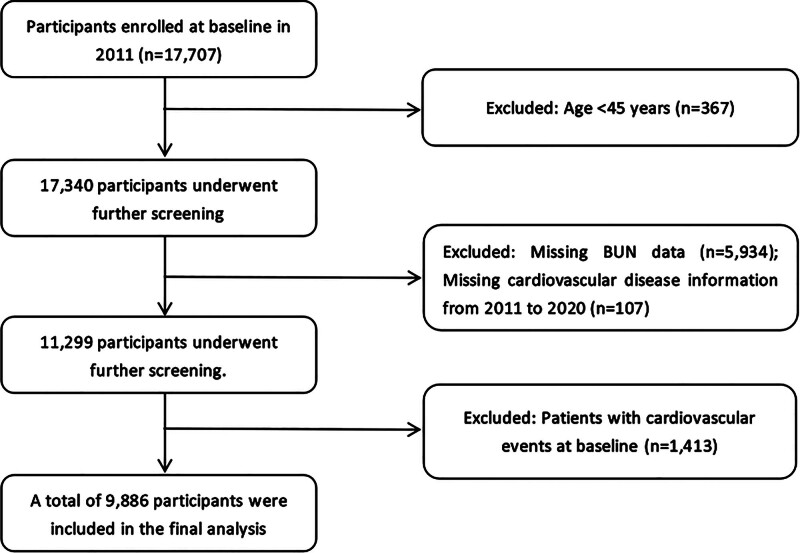
Flowchart of participant selection for the CHARLS Cohort Study, 2011–2020 (n = 9889). CHARLS = China Health and Retirement Longitudinal Study.

The CHARLS is a nationwide longitudinal cohort funded by the National Natural Science Foundation of China. It aims to systematically collect high-quality, nationally representative microdata on Chinese adults aged 45 years and older, serving as a critical resource for research on population aging and interdisciplinary health sciences. Given CHARLS’s strengths in sample representativeness, completeness of follow-up data, and standardized biospecimen collection, this study utilized its data to investigate the potential association between BUN levels and the risk of CVD. The CHARLS project was initiated in 2011 and conducts follow-up surveys every 2 to 3 years. It covers 28 provinces, 150 counties, and 450 communities or villages across China, involving more than 10,000 households. In the 2011 baseline survey, fasting venous blood samples were collected from participants who had fasted for at least 12 hours. BUN concentration was measured using a standardized enzymatic colorimetric method, with all biochemical analyses performed by certified laboratory technicians following uniform protocols. Figure 1 illustrates participants. A total of 17,707 individuals completed physical examinations and questionnaire assessments at baseline. Based on the study design, the following participants were excluded: 367 individuals younger than 45 years, 5934 individuals with missing BUN data, 107 individuals lacking information on CVD status, and 1413 individuals who had already been diagnosed with CVD at baseline.

### 2.2. Content of the study

#### 2.2.1. General information collection

This study, based on data from CHARLS, aims to investigate the association between BUN levels and the risk of CVD among middle-aged and older adults aged 45 years and above in China. Ultimately, 9886 participants were included in the final analysis. The data was collected by medically trained interviewers through face-to-face interviews using a meticulously designed, standardized, and structured questionnaire.

On this basis, multiple covariates were included for analysis to control potential confounding factors. These covariates were categorized into 4 groups: demographic characteristics, lifestyle factors, current health conditions, and laboratory indicators. Demographic characteristics included gender, age, education level (primary school or below/ junior high school/ high school or above), residence (urban/ rural), and marital status (married/ living alone). Lifestyle factors included sleep duration, smoking status (smoker/ nonsmoker), alcohol consumption frequency (nondrinker/ less than once per month/ more than once per month), and meal frequency, which was categorized as frequent (≥4 times/d), normal (3 times/d), or infrequent (≤2 times/d). Health conditions included hypertension and diabetes. Diabetes was defined according to the 2005 American Diabetes Association criteria as fasting glucose ≥ 126 mg/dL, random glucose ≥ 200 mg/dL, HbA1c ≥ 6.5%, or self-reported diagnosis.^[15]^ Hypertension was defined based on the 2020 International Society of Hypertension Global Practice Guidelines as systolic blood pressure ≥ 140 mm Hg and/or diastolic blood pressure ≥ 90 mm Hg, or self-reported previous diagnosis.^[16]^ Laboratory indicators included body mass index (BMI), categorized according to Chinese adult standards as underweight (<18.5), normal (18.5–23.9), overweight (24–27.9), and obese (≥28).^[17]^

#### 2.2.2. Baseline variables

This study utilized the measurement values of BUN obtained from the baseline survey of the CHARLS in 2011, with BUN concentration as the primary exposure variable. To improve the accuracy of the analysis and minimize the influence of outliers or potential measurement errors, only samples with BUN values within the range of 5 to 35 mg/dL were included. All BUN data were obtained from fasting venous blood samples and measured using a standardized enzymatic colorimetric method in certified laboratories. These baseline BUN levels were used to assess their association with the occurrence rate of CVD during the follow-up period.

#### 2.2.3. Ending variables

To identify incident CVD events, this study relied on self-reported physician diagnoses collected during follow-up interviews. Participants were asked whether they had ever been diagnosed by a doctor with heart disease, including myocardial infarction, coronary heart disease, angina, congestive heart failure, or other heart conditions. Those who responded affirmatively were classified as having CVD. Individuals with a history of CVD at baseline (2011) were excluded to ensure the cohort represented a population without preexisting cardiovascular conditions. Participants who first reported a doctor-diagnosed CVD during the follow-up period (up to 2020) were considered to have developed incident CVD. This definition was used to establish the primary outcome variable for analysis, enabling the assessment of the prospective association between baseline BUN levels and CVD onset. To ensure data reliability and accuracy, all interviewers underwent systematic training followed by the implementation of strict quality control measures for data recording and confirmation.

### 2.3. Statistical methods

This study was based on data from CHARLS collected between 2011 and 2020, ultimately including 9886 eligible participants. All participants provided written informed consent, and the study protocol was approved by the Biomedical Ethics Committee of Peking University (approval number: IRB00001052-11015). Continuous variables were summarized using medians and interquartile ranges (Q1–Q3) for non-normally distributed data, while categorical variables were presented as frequencies and percentages. Participants were stratified into quartiles according to BUN concentrations to examine baseline differences. Group comparisons were conducted using χ^2^ tests for categorical variables, Kruskal–Wallis *H* tests for non-normally distributed continuous variables, and one-way ANOVA for normally distributed variables. To investigate the association between BUN levels and incident CVD, 3 logistic regression models were constructed, with results reported as odds ratios (ORs) and 95% confidence intervals (CIs). Model 1 was unadjusted; Model 2 adjusted for demographic characteristics, including age, sex, education level, residence, and marital status; and Model 3 further accounted for health behaviors (e.g., smoking, alcohol consumption, sleep duration) and laboratory indicators. Potential nonlinear relationships between BUN and CVD risk were assessed using restricted cubic spline (RCS) regression with knots placed at the 5th, 35th, 65th, and 95th percentiles of BUN distribution. The dose–response relationship was visualized using spline plots. Additionally, to examine whether the association between BUN and CVD risk was modified by sociodemographic factors or health behaviors, interaction terms were introduced into the regression models. The statistical significance of these interactions was evaluated using likelihood ratio tests.

All missing data were performed using R software (version 4.4.1). Multiple interpolations were performed using the “VIM” and Multivariate Imputation by Chained Equations’ packages. All models were adjusted for relevant covariates, and statistical analyses were performed using R software (version 4.4.1), with sample interview weights applied to account for the survey design. Statistical significance was defined as a *P*-value < .05.

To evaluate the robustness of our findings, we conducted 2 sensitivity analyses: one using the original dataset with missing values, which underwent multiple imputation via the Multivariate Imputation by Chained Equations method (5 imputation rounds, with baseline characteristics and primary outcome variables included in the imputation model); and another using complete case analysis (including only participants without missing data). The robustness of the results was assessed by comparing the effect estimates of the primary outcome and the consistency of between-group differences in baseline characteristics (*P*-values) across both analytical approaches.

## 3. Results

### 3.1. Basic situation analysis

Table 1 summarizes the baseline characteristics of the study population stratified by incident CVD status during follow-up. Among the 9886 participants included, 1055 (10.67%) developed CVD by 2020, while 8831 (89.33%) did not. Compared with participants without CVD, those who developed CVD had a higher prevalence of chronic conditions such as hypertension and diabetes, and were more likely to report smoking and alcohol consumption. Additionally, they exhibited shorter average sleep duration and lower BMI, suggesting that both lifestyle and health conditions may differ substantially between the 2 groups. Sex differences were also observed, with a significantly higher proportion of females among CVD cases compared to non-CVD cases (*P* < .001), indicating a potential sex-related disparity in CVD incidence.Interestingly, baseline BUN levels were significantly lower in the CVD group than in the non-CVD group (*P* = .005). Although the direction of this association was unexpected, it highlights the need for further research to clarify the potential role of BUN in CVD pathogenesis.

**Table 1:** Extreme characteristics of the population with cardiovascular disease based on follow-up [M (P25, P75), n(%)].

Variables	Total (n = 9886)	NO-CVD (n = 8831)	CVD (n = 1055)	Statistic	*P*
BUN, M (Q1, Q3)	15.15 (12.58, 18.23)	15.18 (12.60, 18.26)	14.87 (12.39, 17.67)	*Z* = −2.78	**.005**
Age, M (Q1, Q3)	58.00 (51.00, 65.00)	58.00 (51.00, 65.00)	59.00 (53.00, 64.00)	*Z* = −0.95	.344
Sleeptime, M (Q1, Q3)	6.50 (5.00, 8.00)	7.00 (5.00, 8.00)	6.00 (5.00, 8.00)	*Z* = −2.79	**.005**
BMI, M (Q1, Q3)	23.08 (20.80, 25.64)	22.98 (20.72, 25.48)	24.13 (21.77, 26.85)	*Z* = −9.58	**<.001**
Sex, n (%)					
Female	5123 (51.82)	4472 (50.64)	651 (61.71)	χ^2^ = 46.23	**<.001**
Male	4763 (48.18)	4359 (49.36)	404 (38.29)		
Marital, n (%)					
Living alone	1153 (11.66)	1057 (11.97)	96 (9.10)	χ^2^ = 7.53	**.006**
Marriage	8733 (88.34)	7774 (88.03)	959 (90.90)		
Education, n (%)					
Junior High School	2002 (20.25)	1779 (20.14)	223 (21.14)	χ^2^ = 1.53	.466
High School education or above	1051 (10.63)	931 (10.54)	120 (11.37)		
Primary School and below	6833 (69.12)	6121 (69.31)	712 (67.49)		
Location, n (%)					
City	9111 (92.16)	8137 (92.14)	974 (92.32)	χ^2^ = 0.04	.836
Rural area	775 (7.84)	694 (7.86)	81 (7.68)		
Smoking, n (%)					
Do not smoke	5947 (60.16)	5252 (59.47)	695 (65.88)	χ^2^ = 16.13	**<.001**
Smoking	3939 (39.84)	3579 (40.53)	360 (34.12)		
Drinking, n (%)					
Drink but less than once a month	801 (8.10)	719 (8.14)	82 (7.77)	χ^2^ = 12.74	**.002**
Drink more than once a month	2597 (26.27)	2366 (26.79)	231 (21.90)		
None of these	6488 (65.63)	5746 (65.07)	742 (70.33)		
Number of meals, n (%)					
Multiple meals	139 (1.41)	124 (1.40)	15 (1.42)	χ^2^ = 1.08	.582
Frequent meals	1320 (13.35)	1190 (13.48)	130 (12.32)		
Normal meals	8427 (85.24)	7517 (85.12)	910 (86.26)		
Diabetes, n (%)					
No	8254 (83.49)	7403 (83.83)	851 (80.66)	χ^2^ = 6.85	**.009**
Yes	1632 (16.51)	1428 (16.17)	204 (19.34)		
Hypertension, n (%)					
No	6046 (61.16)	5496 (62.24)	550 (52.13)	χ^2^ = 40.49	**<.001**
Yes	3840 (38.84)	3335 (37.76)	505 (47.87)		

*P* values were derived from χ^2^ tests for categorical variables, rank-sum tests for continuous variables with non-normal distribution, and *t*-tests for continuous variables with normal distribution. The bold values denote statistical significance.

BMI = body mass index, BUN = blood urea nitrogen, CVD = cardiovascular disease, NO-CVD = no cardiovascular disease.

### 3.2. Characteristics of the study population according to BUN quartiles

Table 2 presents the baseline characteristics of participants stratified by quartiles of BUN levels. The incidence of newly diagnosed CVD progressively declined across increasing BUN quartiles, with rates of 11.54%, 11.25%, 10.65%, and 9.26%, respectively. This trend suggests an inverse association between BUN levels and CVD risk, indicating that lower BUN concentrations may be linked to increased CVD incidence in middle-aged and older adults. Notably, significant differences were observed in dietary behavior, particularly meal frequency, across BUN quartiles (*P* < .001). Among participants with low meal frequency (≤2 meals/d), CVD incidence increased with higher BUN levels – 11.78%, 12.59%, 13.02%, and 16.01%, respectively. In contrast, individuals reporting a standard meal frequency (3 meals/d) had consistently lower CVD incidence. These findings imply that meal frequency may modify the relationship between BUN levels and CVD risk. Furthermore, several baseline characteristics varied significantly across BUN quartile groups (all *P* < .001), including age, sex, BMI, educational attainment, residence, smoking status, and alcohol consumption. These variables may act as confounding or interacting factors and were therefore included in multivariable models to more accurately evaluate the independent association between BUN levels and CVD risk.

**Table 2:** Baseline characteristics of the study population according to blood urea nitrogen quartiles [M (P25, P75), n(%)].

Variables	Total (n = 9886)	Q1 (n = 2461)	Q2 (n = 2463)	Q3 (n = 2489)	Q4 (n = 2473)	Statistic	*P*
Age, M (Q1, Q3)	58.00 (51.00, 65.00)	56.00 (49.00,63.00)	57.00 (51.00, 64.00)	58.00 (52.00, 65.00)	60.00 (54.00, 68.00)	χ^2^ = 204.51*	**<.001**
Sleeptime, M (Q1, Q3)	6.50 (5.00, 8.00)	7.00 (5.00, 8.00)	7.00 (5.00, 8.00)	6.50 (5.00, 8.00)	6.00 (5.00, 8.00)	χ^2^ = 5.33*	.149
BMI, M (Q1, Q3)	23.08 (20.80, 25.64)	23.31 (21.10, 25.92)	23.27 (20.93, 25.95)	23.21 (20.82, 25.71)	22.55 (20.40, 25.01)	χ^2^ = 70.17*	**<.001**
Sex, n (%)							
Female	5123 (51.82)	1543 (62.70)	1378 (55.95)	1204 (48.37)	998 (40.36)	χ^2^ = 275.48	**<.001**
Male	4763 (48.18)	918 (37.30)	1085 (44.05)	1285 (51.63)	1475 (59.64)		
Marital, n (%)							
Living alone	1153 (11.66)	265 (10.77)	288 (11.69)	283 (11.37)	317 (12.82)	χ^2^ = 5.33	.149
Marriage	8733 (88.34)	2196 (89.23)	2175 (88.31)	2206 (88.63)	2156 (87.18)		
Education, n (%)							
Junior High School	2002 (20.25)	499 (20.28)	564 (22.90)	500 (20.09)	439 (17.75)	χ^2^ = 33.81	**<.001**
High School education or above	1051 (10.63)	281 (11.42)	288 (11.69)	240 (9.64)	242 (9.79)		
Primary School and below	6833 (69.12)	1681 (68.31)	1611 (65.41)	1749 (70.27)	1792 (72.46)		
Location, n (%)							
City	9111 (92.16)	2237 (90.90)	2248 (91.27)	2310 (92.81)	2316 (93.65)	χ^2^ = 17.18	**<.001**
Rural area	775 (7.84)	224 (9.10)	215 (8.73)	179 (7.19)	157 (6.35)		
Smoking, n (%)							
Do not smoke	5947 (60.16)	1663 (67.57)	1560 (63.34)	1438 (57.77)	1286 (52.00)	χ^2^ = 141.40	**<.001**
Smoking	3939 (39.84)	798 (32.43)	903 (36.66)	1051 (42.23)	1187 (48.00)		
Drinking, n (%)							
Drink but less than once a month	801 (8.10)	191 (7.76)	215 (8.73)	188 (7.55)	207 (8.37)	χ^2^ = 88.75	**<.001**
Drink more than once a month	2597 (26.27)	525 (21.33)	582 (23.63)	707 (28.40)	783 (31.66)		
No drinking	6488 (65.63)	1745 (70.91)	1666 (67.64)	1594 (64.04)	1483 (59.97)		
Number of meals, n (%)							
Multiple meals	139 (1.41)	39 (1.58)	24 (0.97)	40 (1.61)	36 (1.46)	χ^2^ = 26.63	**<.001**
Frequent meals	1320 (13.35)	290 (11.78)	310 (12.59)	324 (13.02)	396 (16.01)		
Normal meals	8427 (85.24)	2132 (86.63)	2129 (86.44)	2125 (85.38)	2041 (82.53)		
CVD, n (%)							
No	8831 (89.33)	2177 (88.46)	2186 (88.75)	2224 (89.35)	2244 (90.74)	χ^2^ = 7.97	.047
Yes	1055 (10.67)	284 (11.54)	277 (11.25)	265 (10.65)	229 (9.26)		
Diabetes, n (%)							
No	8254 (83.49)	2089 (84.88)	2062 (83.72)	2070 (83.17)	2033 (82.21)	χ^2^ = 6.70	.082
Yes	1632 (16.51)	372 (15.12)	401 (16.28)	419 (16.83)	440 (17.79)		
Hypertension, n (%)							
No	6046 (61.16)	1530 (62.17)	1499 (60.86)	1528 (61.39)	1489 (60.21)	χ^2^ = 2.14	.543
Yes	3840 (38.84)	931 (37.83)	964 (39.14)	961 (38.61)	984 (39.79)		

*P* values were calculated using the χ^2^ test for categorical variables, the rank-sum test for non-normally distributed continuous variables, or analysis of variance (ANOVA) for normally distributed continuous variables. The bold values denote statistical significance.

CVD = cardiovascular disease.

* Rank-sum test was used.

### 3.3. Association between baseline BUN and new-onset CVD

Table 3 presents the results of the multivariable logistic regression analyses. Using the lowest quartile (Q1) of BUN as the reference, participants in the highest quartile (Q4) exhibited a 15% lower odds of developing CVD in Model 2 (OR = 0.85, 95% CI: 0.71–1.03), which adjusted for demographic characteristics. In Model 3, which further adjusted for health behaviors and laboratory indicators, the association became slightly stronger, with a 17% lower odds of CVD (OR = 0.83, 95% CI: 0.69–1.01). Although the associations did not reach conventional statistical significance, a marginally significant inverse trend was observed across increasing BUN quartiles (*P* for trend = .081). These findings suggest a possible inverse relationship between BUN levels and CVD risk, highlighting the potential utility of BUN as a biomarker for cardiovascular risk assessment in middle-aged and older adults. In addition, Table 4 presents a trend test for the quartile groups of BUN in the Cox model. The results indicate that as BUN levels increase from Q1 to Q4, the cumulative risk of CVD shows a gradually decreasing trend. Although this did not reach conventional statistical significance (*P* < .05), it is highly consistent with the marginally significant negative trend observed in the logistic regression analysis (*P* for trend = .081), further confirming the directional stability of the association between BUN and CVD risk.

**Table 3:** Multivariate logistic regression analyses of blood urea nitrogen and cardiovascular disease.

	Model 1	*P*	Model 2	*P*	Model 3	*P*
BUN IQR	0.886 (0.813, 0.965)	.005	0.928 (0.85, 1.012)	.091	0.914 (0.837, 0.998)	.045
Q1	Ref		Ref		Ref	
Q2	0.96 (0.80, 1.14)	.631	0.98 (0.82, 1.17)	.811	0.98 (0.82, 1.17)	.849
Q3	0.92 (0.77, 1.10)	.357	0.98 (0.82, 1.17)	.813	0.97 (0.81, 1.16)	.755
Q4	0.77 (0.64, 0.93)	.007	0.85 (0.71, 1.03)	.102	0.83 (0.69, 1.01)	.064
	<0.007		0.132		0.081	

Q1 = Quartile 1; Q2 = Quartile 2 (median); Q3 = Quartile 3; Q4 = Quartile 4.

Model 1: Crude; Model 2: Adjust: age, sex, marital, education, location, smoking, drinking; Model 3: Adjust: age, sex, marital, education, location, smoking, drinking, sleeptime, number of meals, BMI, diabetes, hypertension.

BMI = body mass index, BUN = blood urea nitrogen, CI = confidence interval, IQR = per interquartile range, OR = odds ratio.

**Table 4:** Cox regression analyses of blood urea nitrogen and cardiovascular disease.

	Model 1	*P*	Model 2	*P*	Model 3	*P*
BUN IQR	0.980 (0.966, 0.994)	.005	0.988 (0.973, 1.002)	.094	0.988 (0.974, 1.003)	.112
Q1	Ref		Ref		Ref	
Q2	0.960 (0.814, 1.133)	.631	0.980 (0.83, 1.157)	.811	0.968 (0.82, 1.143)	.705
Q3	0.925 (0.783, 1.092)	.358	0.982 (0.83, 1.162)	.837	0.970 (0.82, 1.148)	.726
Q4	0.786 (0.660, 0.935)	.007	0.863 (0.722, 1.032)	.106	0.868 (0.726, 1.038)	.121
	0.007		0.140		0.153	

Q1 = Quartile 1; Q2 = Quartile 2 (median); Q3 = Quartile 3; Q4 = Quartile 4.

Model 1: Crude; Model 2: Adjust: Age, sex, marital, education, location, smoking, drinking; Model 3: Adjust: Age, sex, marital, education, location, smoking, drinking, sleep time, number of meals, BMI, diabetes, hypertension.

BMI = body mass index, BUN = blood urea nitrogen, CI = confidence interval, HR = hazard ratio, IQR = per interquartile range.

### 3.4. Dose-response relationship between BUN and CVD

**Figure 2.**
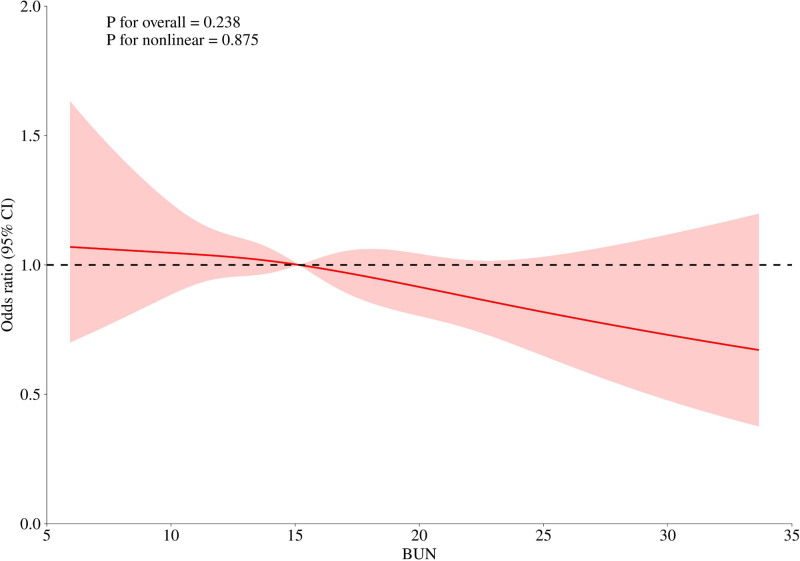
Dose-response relationship between BUN and CVD [OR (95% CI)]. BUN = blood urea nitrogen, CI = confidence interval, CVD = cardiovascular diseases, OR = odds ratio.

Figure 2 illustrates the association between BUN levels and the risk of incident CVD, modeled using a RCS function. After full adjustment for potential confounders, no statistically significant nonlinear relationship was observed (*P* for nonlinearity = .0879), although the overall association reached marginal significance (*P* for overall association = .0238). A potential inflection point was identified at a BUN concentration of 15.13 mg/dL, where the estimated OR was equal to 1. Below this threshold, increasing BUN levels were associated with a higher risk of CVD; conversely, above this threshold, higher BUN levels were associated with a decreased risk of CVD. These findings suggest a possible U-shaped or threshold-dependent relationship between BUN and CVD risk.

### 3.5. Subgroup analysis

**Figure 3.**
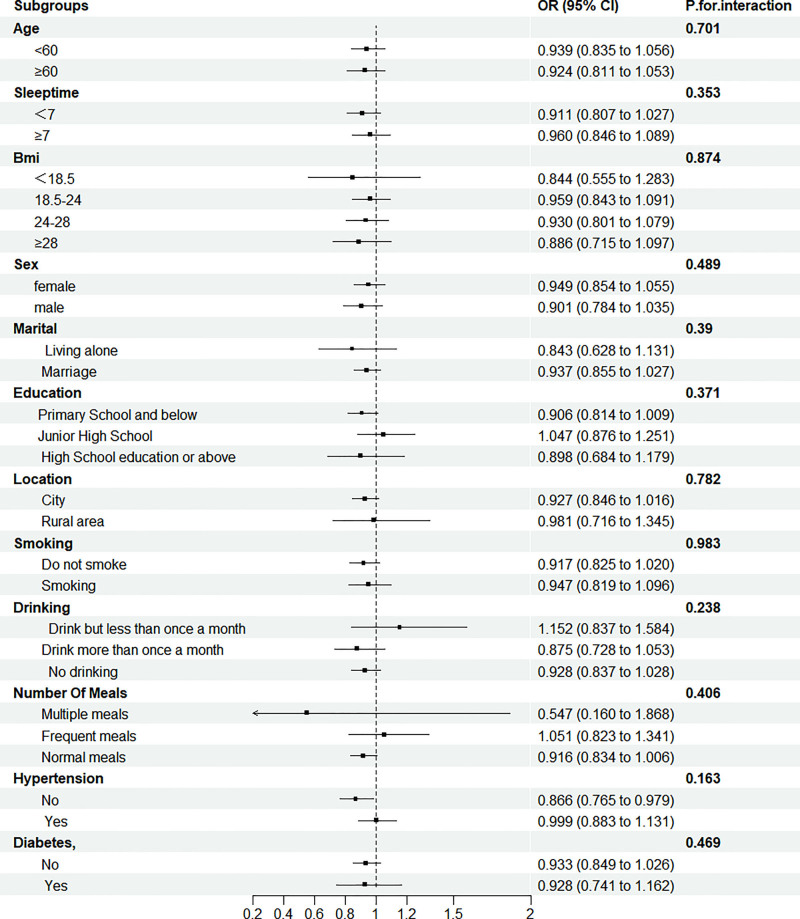
Subgroup analysis and forest plot of the association between BUN and CVD Incidence. BUN = blood urea nitrogen, CVD = cardiovascular disease.

To assess the robustness of the association between BUN levels and incident CVD, subgroup analyses were conducted. Participants were stratified based on sociodemographic characteristics (e.g., sex, age, educational level) and baseline comorbidities (e.g., hypertension, diabetes), and the association between BUN levels and CVD risk was evaluated within each subgroup. Interaction terms were included in the models to test for potential effect modification. As shown in Figure 3, no statistically significant interactions were observed between BUN levels and any of the stratifying variables (all *P* for interaction > 0.05), suggesting that the association between BUN and CVD risk was consistent across subpopulations. These findings support the robustness and potential generalizability of the observed relationship.

## 4. Discussion

Using data from the 2011 wave of CHARLS, this study included middle-aged and older adults (aged ≥ 45 years) who were free of CVD at baseline and conducted a 9-year prospective follow-up to examine the association between BUN levels and incident CVD. A total of 9886 participants met the inclusion and exclusion criteria, among whom 1055 new cases of CVD were identified during the follow-up period. The findings demonstrated an inverse association between BUN levels – within a certain range – and the risk of developing CVD. This relationship remained consistent across multivariable-adjusted logistic regression models. Dose–response analysis using RCS modeling revealed a nonlinear trend (*P* for overall association = .0238; *P* for nonlinearity = .0879), with a threshold effect observed at a BUN level of 15.13 mg/dL. Specifically, the risk of CVD decreased as BUN levels exceeded this threshold, indicating a potential protective effect. This protective association may be explained by the role of moderately elevated BUN in reflecting better nutritional and renal functional status, which could contribute to improved metabolic homeostasis and reduced cardiovascular burden. However, given that this negative linear relationship only demonstrated borderline statistical significance, the result should be interpreted as a suggestive trend rather than a definitive association. Further research is warranted to validate and explore this potential relationship. In conclusion, BUN – an inexpensive, routinely measured biomarker – may have clinical utility in predicting cardiovascular risk and guiding early prevention strategies among middle-aged and older adults.

Previous studies have established a close association between BUN levels and the development of CVD, particularly among high-risk populations. Most of these studies have emphasized the deleterious effects of elevated BUN levels on cardiovascular outcomes, with proposed mechanisms including the upregulation of pro-apoptotic proteins, inhibition of microvascular endothelial cell proliferation, and promotion of atherosclerosis progression.^[11–13,18]^ For instance, the Dongfeng-Tongji prospective cohort study reported a significant positive association between higher BUN levels and the risk of coronary heart disease.^[19]^ Similarly, a large-scale study involving 26,835 Chinese adults identified a nonlinear relationship between BUN levels and CVD mortality, suggesting that both abnormally low and high BUN levels may elevate the risk of stroke-related death.^[20]^ However, the optimal physiological range of BUN, particularly in the general middle-aged and older adult population, remains insufficiently defined. In contrast to earlier findings, the present study observed that lower BUN levels were associated with a higher risk of incident CVD, suggesting a potential bidirectional role of BUN in cardiovascular regulation. This counterintuitive association may be partly attributable to suboptimal nutritional status and inadequate dietary patterns prevalent among older adults in China. Specifically, in certain regions, poor diet quality – characterized by low protein intake and insufficient antioxidant nutrients – may contribute to endothelial dysfunction through mechanisms such as gut microbiota dysbiosis, chronic inflammation, and lipid peroxidation, ultimately promoting CVD development.^[21]^ According to the 2024 Chinese Expert Consensus on Home Nutrition Management for Elderly Patients,^[22]^ the combined prevalence of malnutrition and its risk among community-dwelling older adults in China is as high as 41.2%, with rural populations disproportionately affected. These findings underscore the importance of identifying an appropriate physiological range of BUN as a potential marker for early CVD risk detection. Based on our study, BUN levels in the range of 15.13 to 35 mg/dL may confer cardiovascular protection, offering novel insights into the role of nutritional status in CVD prevention and informing targeted interventions for aging populations.

Clinical consensus suggests that BUN levels are influenced by a range of external factors, with dietary structure and meal frequency playing particularly critical roles in modulating BUN concentrations.^[23]^ In the present study, we observed that a low meal frequency (≤2 meals per day) was significantly associated with a higher risk of incident CVD, indicating that irregular eating patterns may disrupt metabolic homeostasis and impose additional stress on the cardiovascular system. Physiologically, during prolonged fasting states, the body’s overall metabolic rate declines, resulting in reduced hepatic urea synthesis capacity.^[24]^ In middle-aged and older adults, this phenomenon is further exacerbated by age-related declines in basal metabolic rate, which may impair the activity of key enzymes in the urea cycle and contribute to lower circulating BUN levels. On the other hand, acute consumption of excessive dietary protein can rapidly elevate BUN levels. This high-BUN state may induce chronic low-grade inflammation and endothelial dysfunction through mechanisms such as protein carbamylation and increased production of ROS, thereby heightening CVD risk.^[25]^ To maintain metabolic stability and support cardiovascular health, it is essential for middle-aged and older adults to undergo regular assessments of dietary protein intake, total caloric consumption, and micronutrient adequacy.^[26]^ Promoting dietary diversity – especially with increased intake of antioxidant-rich vitamins and minerals – may help delay vascular aging, mitigate systemic inflammation, and confer long-term cardiovascular benefits in aging populations.^[27]^

This study, based on the nationally representative CHARLS cohort, systematically examined the association between BUN levels and incident CVD, with notable strengths including a large sample size and an extended follow-up period. However, several limitations should be acknowledged. First, regarding data completeness, the CHARLS survey did not systematically collect detailed information on dietary composition, particularly with respect to high-protein dietary patterns, such as the intake proportions of red meat versus plant-based proteins. In addition, data on the use of medications known to affect BUN metabolism – such as antibiotics, diuretics, and glucocorticoids – were unavailable. As a result, our ability to comprehensively adjust for potential confounders was limited, which may have influenced the precision of the observed associations and the ability to infer causality.Second, in terms of outcome ascertainment, CVD events in CHARLS were identified exclusively through self-reported physician diagnoses (i.e., “Has a doctor ever told you that you have heart disease?”), rather than through standardized diagnostic criteria such as International Classification of Diseases codes. This limitation precluded differentiation among specific CVD subtypes (e.g., myocardial infarction, heart failure, ischemic stroke), thereby restricting our capacity to explore subtype-specific associations and dose–response relationships with BUN levels. Furthermore, as an observational study based on self-reported survey data, CHARLS is subject to several types of measurement bias. These include diagnostic bias due to unequal access to healthcare services, recall bias associated with age-related cognitive decline, and reporting bias linked to individual differences in health literacy. Such systematic biases are difficult to fully eliminate through statistical adjustment and may affect the validity and generalizability of the findings. In conclusion, while this study provides important preliminary insights into the relationship between BUN levels and CVD risk, future research should incorporate more detailed dietary and pharmacologic data, utilize clinically validated outcome measures, and leverage high-quality multicenter biological data to elucidate the causal pathways and underlying mechanisms involved.

## 5. Conclusion

In conclusion, this study highlights a significant association between BUN levels and the incidence of CVD among middle-aged and older adults – though it should be noted that the association only reached borderline statistical significance, so the current research results should be regarded as preliminary findings. As a routinely measured and readily accessible clinical biomarker, BUN may have potential utility in the early identification of individuals at elevated risk for CVD. However, in clinical settings, it is crucial to interpret BUN levels within a comprehensive clinical context to avoid overdiagnosis or misclassification resulting from mild or transient elevations. Furthermore, our findings underscore the importance of maintaining regular eating patterns and ensuring adequate intake of high-quality dietary protein among older adults, which may help support nutritional and metabolic homeostasis and, in turn, reduce cardiovascular risk. Given the observational nature of this study, causality cannot be definitively established. Therefore, it is necessary to carry out large-scale, multi-center prospective studies in the future to verify these findings, and to further explore the biological mechanisms and potential interventional implications of BUN in cardiovascular health management.

## Acknowledgments

We thank CHARLS for providing nationally representative data for robust analyses. We thank every respondent for their time and efforts that they have devoted to the CHARLS project. Thanks are also extended to every colleague who participants in the study.

## Author contributions

**Conceptualization:** Renyu Jiang, Jinyan Chen.

**Data curation:** Qi Wang.

**Formal analysis:** Renyu Jiang.

**Funding acquisition:** Yong Hu.

**Investigation:** Qi Wang, Yong Hu.

**Methodology:** Renyu Jiang.

**Project administration:** Jinyan Chen, Yong Hu.

**Software:** Qi Wang.

**Validation:** Renyu Jiang, Yong Hu.

**Visualization:** Qi Wang, Jinyan Chen.

**Writing** – **original draft:** Renyu Jiang, Jinyan Chen.

**Writing** – **review & editing:** Yong Hu.
